# Hyaluronidase Inhibitor-Incorporated Cross-Linked Hyaluronic Acid Hydrogels for Subcutaneous Injection

**DOI:** 10.3390/pharmaceutics13020170

**Published:** 2021-01-27

**Authors:** Min-Hwan Kim, Ju-Hwan Park, Duy-Thuc Nguyen, Sungyun Kim, Da In Jeong, Hyun-Jong Cho, Dae-Duk Kim

**Affiliations:** 1 College of Pharmacy and Research Institute of Pharmaceutical Sciences, Seoul National University, Seoul 08826, Korea; mhkim305@snu.ac.kr (M.-H.K.); parkjh0116@snu.ac.kr (J.-H.P.); nguyenduythuc92@snu.ac.kr (D.-T.N.); 2 College of Pharmacy, Kangwon National University, Chuncheon, Gangwon 24341, Korea; sungyun@kangwon.ac.kr (S.K.); jdi0327@kangwon.ac.kr (D.I.J.)

**Keywords:** cross-linking, hyaluronic acid, hyaluronidase inhibitor, hydrogel, quetiapine, sustained release

## Abstract

Hyaluronidase (HAase) inhibitor-incorporated hyaluronic acid (HA) hydrogel cross-linked with 1,4-butanediol diglycidyl ether (BDDE) was designed to reduce the toxicity risk induced by BDDE and its biodegradation rate in subcutaneous tissue. The formulation composition of hydrogel and its preparation method were optimized to have a high swelling ratio and drug content. Quercetin (QCT) and quetiapine (QTP), as an HAase inhibitor and model drug, respectively, were incorporated into the cross-linked hydrogel using the antisolvent precipitation method for extending their release after subcutaneous injection. The cross-linked HA (cHA)-based hydrogels displayed appropriate viscoelasticity and injectability for subcutaneous injection. The incorporation of QCT (as an HAase inhibitor) in the cHA hydrogel formulation resulted in slower in vitro and in vivo degradation profiles compared to the hydrogel without QCT. Single dosing of optimized hydrogel injected via a subcutaneous route in rats did not induce any acute toxicities in the blood chemistry and histological staining studies. In the pharmacokinetic study of rats following subcutaneous injection, the cHA hydrogel with QCT exhibited a lower maximum QTP concentration and longer half-life and mean residence time values compared to the hydrogel without QCT. All of these results support the designed HAase inhibitor-incorporated cHA hydrogel being a biocompatible subcutaneous injection formulation for sustained drug delivery.

## 1. Introduction

Hyaluronic acid (HA) has been extensively studied for biomedical applications, including drug delivery, tissue engineering, and cosmetics (e.g., dermal fillers) [1,2,3,4,5,6,7,8]. HA is composed of D-glucuronic acid and *N*-acetyl-D-glucosamine, linked by β-1,3- and β-1,4-glycosidic bonds, and is found in epithelial and connective tissues [9]. It is produced in the plasma membrane by hyaluronan synthase and moved to the extracellular matrix. Some portions of HA are degraded by hyaluronidase (HAase) and other parts can be catabolized, and thus, it shows half-lives of a couple of minutes to weeks in the body [9]. Endo-β-*N*-acetyl hexosaminidase is a major mammalian HAase that can cleave β-1,4-glycosidic bonds in HA [10,11]. Because of its high molecular weight and water absorption capability, HA is essential for the maintenance of the mechanical and viscoelastical roles in the body [9]. These biosynthesis and biodegradation characteristics of HA make it useful for diverse clinical applications, including wound dressings, dermal fillers, and drug delivery.

Moreover, the insufficient mechanical and rheological properties of unmodified HA dispersion have been tuned by physical interactions and chemical reactions [9]. Its anionic charge can be associated with cationic ions, and its carboxylic acid and hydroxyl groups can react with other functional groups for chemical cross-linking [12]. Several chemical reaction strategies (e.g., radical polymerization, click chemistry, disulfide cross-linking, and enzymatic cross-linking) have been employed for designing cross-linked HA (cHA) hydrogels [9]. Among the chemical cross-linkers, which include carbodiimide, divinyl sulfone, hydrazide, methacrylamide, poly(ethylene glycol) diglycidyl ether, and 1,4-butanediol diglycidyl ether (BDDE) [13,14], BDDE has been the most commonly used for making an ether linkage between the hydroxyl group of HA and the epoxide group of BDDE [15,16,17]. As deprotonated hydroxyls (under alkaline conditions) are stronger nucleophiles than the other groups (e.g., the carboxylic acid group) in HA, they can principally participate in the reaction with a cross-linker for forming ether bonds [15]. BDDE is also biodegradable and less toxic than the other cross-linkers for ether bond formation, and thus it has been applied for dermal injection formulations [18]. Therefore, various HA hydrogels cross-linked by BDDE are commercially available in cosmetics, and their application to biomedical engineering and pharmaceutics has been actively investigated [16,17,19]. However, the amount of BDDE in hydrogels needs to be optimized for clinical application for minimizing its toxicity and enhancing its in vivo retention.

The mechanism(s) of drug release from HA-based hydrogels is diverse and complex, which include diffusion/dissolution and erosion as generally shown in other hydrogel delivery systems [20]. HAase inhibitors can keep the balance between the catabolism and anabolism of HA, and various forms of HAase inhibitors (i.e., proteins, glycosaminoglycans, poly/oligosaccharides, fatty acids, and other chemicals) have been reported [10,11]. Thus, the incorporation of HAase inhibitors into HA-based hydrogels would protect the gel degradation by HAase and improve its retention time in injected tissues [21]. Slow degradation of hydrogels could also possibly contribute to the sustained release of the incorporated drug [20]. Several flavonoids have been screened for their HAase inhibitory effects, and quercetin (QCT) has shown a considerable inhibition capacity against bovine testis HAase [22]. Thus, the goal of this study was to design a hydrogel system based on cHA for subcutaneous injection. Cross-linking and preparation methods were optimized to use the minimum amount of BDDE, yet to ensure a high swelling ratio and drug content. This study was focused on the feasibility of reducing the degradation rate of cHA hydrogels by incorporating HAase inhibitors into the hydrogel network. Quetiapine (QTP), as an atypical antipsychotic drug, was loaded into the cHA-based hydrogels, while QCT was employed as an HAase inhibitor for extending the retention period of the hydrogels in the subcutaneous tissue. After optimizing the cHA hydrogel composition and swelling conditions, the antisolvent precipitation method was applied to incorporate both QTP and QCT. To the best of our knowledge, there have been no reports on HAase inhibitor-incorporated cHA hydrogels for retarding the degradation of the hydrogels and extending drug release.

## 2. Materials and Methods

### 2.1. Materials

HA (0.15–0.6 MDa) was provided by SK Bioland Co., Ltd. (Cheonan, Korea). BDDE, HAase (from bovine testes), polyethylene glycol (PEG) 400, and QCT were acquired from Sigma-Aldrich Co. (St. Louis, MO, USA). QTP hemifumarate and sodium lauryl sulfate (SLS) were supplied by Tokyo Chemical Industry (Tokyo, Japan).

### 2.2. Solubility of QTP in Various Media

In order to select a solvent for hydrating (i.e., swelling) the cHA gel, the solubilities of QTP hemifumarate and the QTP base in distilled water (DW), phosphate-buffered saline (PBS; pH = 7.4), and ethanol (EtOH) were determined by high-performance liquid chromatography (HPLC). Briefly, an excess amount of QTP hemifumarate or QTP base was added to 1 mL of each solvent and completely vortex-mixed with vortex shaker (Vortex-Genie 2; Scientific Industries, Inc., Bohemia, NY, USA) at room temperature at 37 °C for 1 day. Upon reaching the steady state, each sample was centrifuged and filtered to eliminate undissolved fractions. An HPLC system equipped with an automatic injector (Waters 717 plus), a pump (Waters 1515; Waters Company, Milford, MA, USA), and a UV/VIS detector (Waters 2487) connected with a reverse-phase C18 column (Kinetex C18; 250 × 4.6 mm, 5 μm; Phenomenex, Torrance, CA, USA) was utilized for measuring the concentration of QTP in each medium. The flow rate of the mobile phase and the injection volume of the specimen were set to 1.0 mL/min and 20 μL, respectively. The mobile phase consisted of 30 mM ammonium acetate in DW (pH 6.0) and methanol (30:70, *v*/*v*). The absorption wavelength was set to 238 nm for detection and the retention time of QTP was 14 min [23].

The QTP base precipitate was prepared using a previously reported desalting method with a slight modification [24]. Briefly, QTP hemifumarate (10 mg) was solubilized in a 1 N HCl solution (0.7 mL), and then, a 1 N NaOH solution (0.8 mL) was added for precipitating the QTP base. The sample was then centrifuged at 16,000× *g* for 5 min and the supernatant portion was eliminated. After adding 1 mL of DW to the precipitate, it was centrifuged again, and the supernatant was removed. Finally, the QTP base precipitate was freeze-dried at −70 °C for 48 h for further use.

### 2.3. Optimization of the Cross-Linking and Swelling Condition of the HA Hydrogels

The epoxy group of BDDE was reacted with the hydroxyl group of HA under alkaline conditions for cross-linking BDDE and HA by an ether bond [25]. The amount of BDDE and the percentage of EtOH for cross-linking and for swelling, respectively, were optimized to maximize the content of QTP in the HA hydrogels. Briefly, HA (50 mg) was dissolved in a 0.25 N NaOH solution (1 mL), followed by mixing with various amounts of BDDE (55, 110, and 165 mg), and then an aliquot (0.1 mL) of each was loaded into each well of a 96-well plate. The cross-linking reaction was carried out at room temperature for 1 day, after which each gel segment was swollen in 10 mL of 0%, 50%, 60%, 70%, and 80% EtOH in DW or PBS for 24 h. Then, they were further incubated in PBS (20 mL) for 24 h. The amount of BDDE and the EtOH percentage-dependent swelling ratios were calculated using the following equation:

Swelling ratio (%) = *W_a_*/*W_b_* × 100, (1)

where *W_a_* and *W_b_* indicate the weight of the hydrogel after and before swelling with PBS for 24 h, respectively.

The content of QTP in the cHA hydrogels (BDDE/HA = 1.1, *w*/*w*) was determined after immersion for 24 h in various percentages (50%, 60%, and 70%) of EtOH in a DW solution (10 mL) containing the QTP base precipitate (100 mg), followed by incubating in PBS (20 mL) for another 24 h. The content of QCT in the cHA hydrogels (BDDE/HA = 1.1, *w*/*w*) containing 10, 30, or 50 mg of QCT (cHA/QCT1, cHA/QCT3, and cHA/QCT5, respectively) was also determined after swelling in EtOH/DW (70:30, *v*/*v*) solution (10 mL) for 24 h, and subsequently incubating in PBS (20 mL) for 24 h.

The QTP and QCT contents in the cHA hydrogels were measured by the HPLC method. Briefly, each hydrogel was placed into the dimethyl sulfoxide and DW (7:3, *v*/*v*) mixture and homogenized by vortex mixing and sonication for 1 h. Then, the mixture was passed through a syringe filter (pore size, 0.20 μm) and QTP and QCT were quantitatively analyzed by HPLC following appropriate dilution with the mobile phase. The HPLC system for QCT and QTP was the same as above, except for the reverse-phase C18 column (Fortis C18; 150 × 4.6 mm, 5 μm; Fortis Technologies Ltd., Cheshire, UK), and the mobile phase consisted of acetonitrile and water (40:60, *v*/*v*), which was used for the QCT analysis. The absorption wavelength and the retention time were 354 nm and 5.6 min, respectively, for QCT [26].

### 2.4. Physicochemical and Rheological Characterization of the cHA Hydrogels

The gelation behavior of the cHA hydrogels was evaluated by various physicochemical and rheological property assessments. In an inversion test, the HA, cHA, cHA/QTP, and cHA/QTP/QCT5 specimens were prepared in plastic tubes and were then inversed for confirming their cross-linking and gelation [27]. The injectability of the cHA hydrogels (i.e., cHA/QTP/QCT5) was investigated by extrusion through a syringe needle (23 gauge). The rheological properties of the hydrogels were studied using an Advanced Rheometric Expansion System with plate–plate geometry (25 mm diameter) [27]. Frequency sweep (1–251 rad/s frequency range and 20% strain) and strain sweep (1–200% strain range and 10 rad/s frequency) measurements were performed at 25 °C. Shear rate-dependent shear stress and viscosity profiles were acquired with modular compact rheometer (MCR 302, Anton Paar GmbH, Graz, Austria) in 0.02–100 s^−1^ of shear rate range.

The in vitro degradation feature of the cHA hydrogel structures was investigated by monitoring the mass change of the hydrogels compared to their initial weight according to a previously reported, slightly modified method [28]. Briefly, to evaluate the weight change of the cHA and QCT-loaded cHA hydrogels (i.e., cHA/QCT1, cHA/QCT3, and cHA/QCT5), each gel specimen (approximately 1.1 g) was placed into 10 mL of a PBS solution containing 5 U/mL of HAase. Upon immersion in the media, the weight of the hydrogels was measured at 2, 4, 6, and 8 h. The remaining weight percentage (%) of each hydrogel compared to the initial weight was recorded.

The release of QTP from the cHA hydrogels was measured using the dialysis bag method [27]. Briefly, aliquots of the free QTP solution (in ethanol/PEG 400/PBS = 1/1/2 (*v*/*v*/*v*)) and the QTP-loaded hydrogels (i.e., cHA/QTP and cHA/QTP/QCT5) (corresponding to 12 mg of QTP) were loaded into dialysis membranes (2 kDa of molecular weight cut-off; Cellu Sep H1, Membrane Filtration Products Ltd., Seguin, TX, USA). In case of cHA/QTP/QCT5 + HAase group, HAase (1 mg/mL) was added to the cHA/QTP/QCT5 hydrogel. Each membrane sac was immersed in PBS (20 mL) containing 0.5% SLS, and then they were agitated in a shaking water bath (37 °C) at 50 rpm. Aliquots of the release media were collected at 0.5, 1, 2, 4, 6, 8, 24, 48, 72, 96, 144, 168, 192, 288, 336, and 360 h, and the same volume of fresh medium was replenished each time. The QTP concentrations in the release media were quantitatively determined by the HPLC method described above.

### 2.5. Biodegradation Study

The biodegradation profiles of the cHA hydrogels (i.e., cHA/QTP and cHA/QTP/QCT5) were observed in the Institute of Cancer Research (ICR) mice (male, 5-weeks-old; Orient Bio, Seongnam, Korea) according to a previously reported method with a slight alteration [29]. The mice were managed in a light-controlled room kept at a temperature of 22 ± 2 °C and a relative humidity of 55 ± 5% (Animal Center for Pharmaceutical Research, College of Pharmacy, Seoul National University, Seoul, Korea). All animal study protocols were approved by the Animal Care and Use Committee of the College of Pharmacy, Seoul National University (SNU-200224-8 and SNU-200703-4). Each hydrogel (0.2 mL) was injected subcutaneously into the mice, and the remaining gel mass was measured after excising on days 2, 4, 7, and 14. The relative remaining weight (%) of the hydrogels was calculated compared to the initial weight.

### 2.6. Systemic Toxicity Studies

The toxicities of the cross-linked hydrogel formulations were assessed by blood chemistry and histology tests [27]. An aliquot (0.2 mL) of HA, cHA, cHA/QCT5, cHA/QTP, and cHA/QTP/QCT5 was injected into the subcutaneous tissue of the dorsal side of the mice, and then, blood samples were obtained via a cardiac puncture on day 7. The concentrations of albumin, alanine transaminase (ALT), aspartate transaminase (AST), and blood urea nitrogen (BUN) in the serum specimens were quantitatively determined with a Cobas 8000 C702 chemical autoanalyzer (Roche Diagnostics, Manheim, Germany). Several organs (i.e., heart, kidneys, liver, lungs, and spleen) were excised from the mice for histological assay. They were incubated in a 4% (*v*/*v*) formaldehyde solution for fixation and tissue slices (~6 μm thickness) were deparaffinized and hydrated with a series of ethanolic solutions. Each specimen was then stained with hematoxylin and eosin (H&E) reagent according to the standard protocol.

### 2.7. In Vivo Pharmacokinetic Study

A pharmacokinetic study of QTP was conducted after subcutaneous injection of the cHA/QTP or cHA/QTP/QCT5 hydrogels (20 mg/kg dose of QTP) in rats. Male Sprague-Dawley (SD) rats were purchased from Orient Bio. The rats were housed in a light-controlled room kept at a temperature of 22 ± 2 °C and a relative humidity of 55 ± 5% (Animal Center for Pharmaceutical Research, College of Pharmacy, Seoul National University). After subcutaneous injection of each hydrogel, aliquots (~250 μL) of blood specimens were collected via a tail vein at 0.5, 1, 2, 4, 6, 8, 24, 48, 72, 120, and 168 h. Following centrifuging at 16,000× *g* for 2.5 min at 4 °C, the supernatant portion (50 μL) was collected and frozen at −20 °C. 

The plasma samples (50 μL) were vortex-mixed with methanol (200 μL) containing carbamazepine (200 ng/mL; internal standard (IS)) for 5 min and centrifuged at 16,000× *g* for 5 min. An aliquot (5 μL) of the supernatant was injected into the liquid chromatography—mass spectrometry/mass spectrometry (LC–MS/MS) system. The Agilent Technologies 1260 Infinity HPLC system (Agilent Technologies, Santa Clara, CA, USA) was equipped with an Agilent Technologies 6430 Triple Quad LC–MS system and connected with a C18 column (Synergi Hydro-RP; 4 μm, 80 Å, 75 mm × 2.0 mm; Phenomenex). The mobile phase was composed of 80% acetonitrile and 20% water containing 5 mM ammonium acetate, and the flow rate was maintained at 0.4 mL/min. The *m/z* values of the precursor to product ion, fragment voltage, collision energy, and cell accelerator voltage for QTP were 384.2 to 253.1, 144 V, 17 eV, and 1 V, respectively. Those for carbamazepine (IS) were 237.0 to 194.0, 126 V, 18 eV, and 1 V, respectively. The retention times of QTP and IS were 1.7 min and 0.7 min, respectively.

The pharmacokinetic parameters (e.g., total area under the plasma concentration-time curve from time zero to infinity (AUC), the peak plasma concentration (C_max_), the time to reach C_max_ (T_max_), half-life (t_1/2_), and mean residence time (MRT)) were acquired via noncompartmental analysis in WinNonlin (ver. 3.1, NCA 201; Pharsight, Mountain View, CA, USA).

### 2.8. Statistical Analysis of the Data

All data were represented as the mean ± standard deviation (SD). Statistical analyses were performed using one-way analysis of variance (ANOVA) and two-tailed Student’s *t*-tests.

## 3. Results and Discussion

### 3.1. Preparation and Optimization of the Cross-Linked Hydrogel Formulations

**Figure 1:**
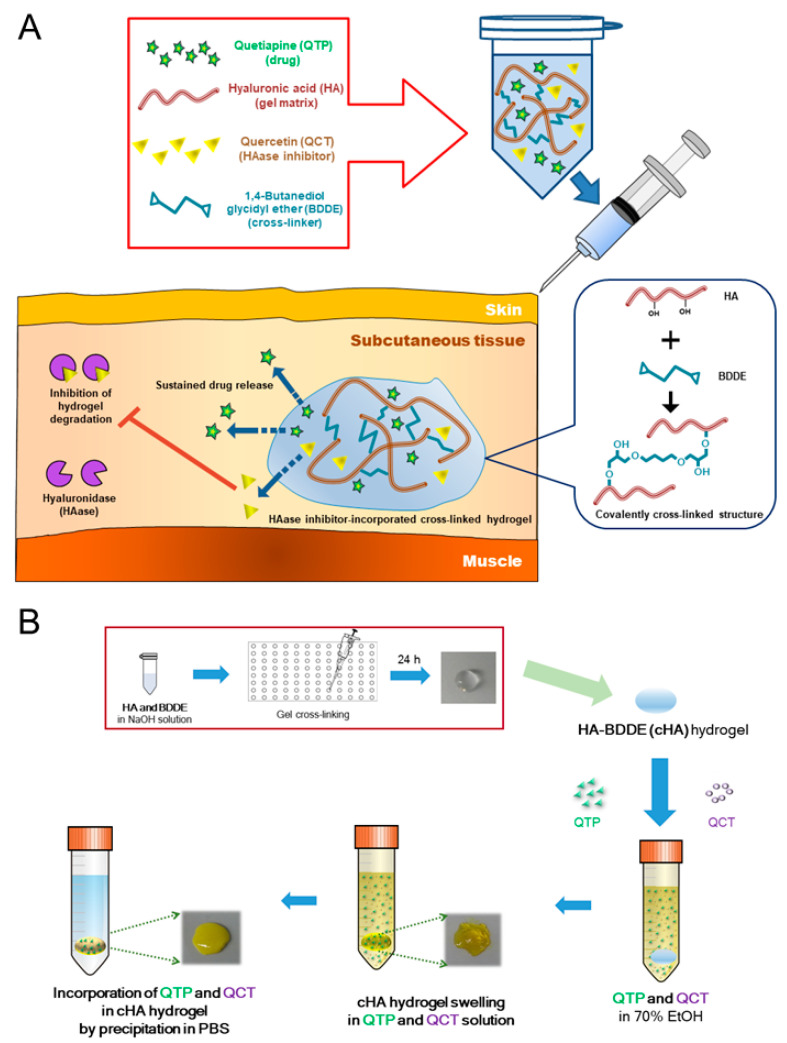
Schematic illustrations of cross-linked hyaluronic acid (cHA)-based hydrogel formulation. (**A**) cHA-based hydrogel formulation strategy for subcutaneous injection. HA was cross-linked with a minimum amount of 1,4-butanediol diglycidyl ether (BDDE) to ensure suitable rheological properties for subcutaneous injection and a high degree of swelling for maximizing the drug content. The incorporation of hyaluronidase (HAase) inhibitors retarded the degradation of cHA, thereby contributing to extending the drug release. (**B**) Preparation process of the cHA-based hydrogels. Incorporation of quetiapine (QTP) and quercetin (QCT), as a model drug and an HAase inhibitor, respectively, in the cHA hydrogels also contributed to sustaining the drug release.

Figure 1A shows a schematic diagram of the cHA-based hydrogel formulations, including the strategy of retarding the degradation of the hydrogels and the sustained release of drug after subcutaneous injection. Figure 1B summarizes the cHA-based hydrogel preparation procedure and incorporation of QCT and QTP by the antisolvent precipitation method. It was reported that QCT was radically grafted to starch backbone and this approach also can be considered as one of the introduction methods of QCT in hydrogel structure [30]. However, the attachment of hydrophobic QCT to hydrophilic HA backbone may alter the hydrogel structure and thus the QCT content could be restricted. 

HA hydrogel has been widely used for commercial dermal fillers [16,17,19], however, the covalent bonding of some other moieties to HA-BDDE may reduce the chances for clinical translation as they will be regarded as new synthetic materials during approval process by regulatory agencies. Therefore, QCT was physically loaded to BDDE-cross-linked HA hydrogels in this study. Under alkaline conditions, the epoxy groups of BDDE were dominantly reacted with the hydroxyl groups (in primary alcohol) of HA, forming an ether bond (Figure S1, Supplementary Materials) [25]. It is known that an ether bond is more stable than ester or amide bond, and thus, fillers based on BDDE-cross-linked HA can show high life expectancy in clinical application [18]. However, a high degree of cross-linking would increase the stiffness and interrupt the injectability through a syringe needle. Thus, we designed cHA hydrogels with minimum BDDE cross-linking to ensure suitable rheological properties for subcutaneous injection and a high degree of swelling for maximizing the drug content. Moreover, the in vivo retention of the cHA hydrogels was extended by incorporating an HAase inhibitor (i.e., QCT in this study) in the cHA hydrogel structure (Figure 1). HAase inhibitors can hinder the degradation process of HA, and thus they are capable of prolonging the retention of HA hydrogels in subcutaneous tissue after injection.

**Figure 2:**
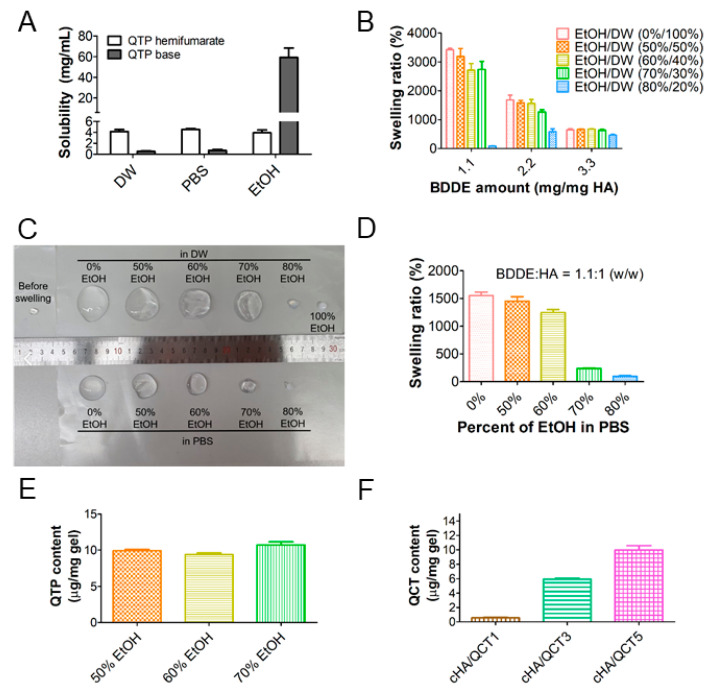
Optimization of the cross-linked hydrogel formulations. (**A**) Solubility of the QTP hemifumarate and QTP base in distilled water (DW), phosphate buffered saline (PBS), and ethanol (EtOH). Each point represents the mean ± SD (*n* = 4). (**B**) Amount of BDDE and EtOH/DW ratio-dependent swelling ratios of the cHA hydrogels. Each point represents the mean ± SD (*n* = 3). (**C**) Images of the cHA hydrogels (BDDE/HA = 1.1, *w*/*w*) before and after 24 h of swelling with various percentages (0%, 50%, 60%, 70%, 80%, and 100%) of EtOH mixed with either DW or PBS. (**D**) Calculated swelling ratios of the cHA hydrogels (BDDE/HA = 1.1, *w*/*w*) after swelling for 24 h with various percentages (0%, 50%, 60%, 70%, and 80%) of EtOH mixed with PBS. Each point represents the mean ± SD (*n* = 3). (**E**) QTP content in the cHA/QTP hydrogels after swelling for 24 h in various percentages (50%, 60%, and 70%) of EtOH in a DW solution, followed by incubation in PBS for 24 h. Each point represents the mean ± SD (*n* = 4). (**F**) QCT content in the cHA/QCT1, cHA/QCT3, and cHA/QCT5 hydrogels. Each point represents the mean ± SD (*n* = 4).

In this study, QTP was incorporated into the hydrogel network as an antipsychotic agent that targets schizophrenia, depressive disorder, and bipolar disorder [31]. As shown in Figure 2A, since the solubility of QTP hemifumarate in DW, PBS, and EtOH was not high enough for the hydrogel preparation, its base precipitate form was prepared by removing the hemifumarate salt. 

The solubility of the QTP base in EtOH was over 50 mg/mL (Figure 2A), and thus, it was selected as a solvent for QTP in the preparation of the hydrogel formulations. Moreover, QCT was practically insoluble in water (<10 µg/mL), but was highly soluble in EtOH (17.64 ± 1.05 mg/mL) (unpublished data), confirming that the EtOH solution was suitable for preparation of cHA hydrogel formulations containing both QTP and QCT. Thus, the preparation of the cross-linking and swelling conditions of the cHA hydrogels was optimized by changing the amount of BDDE and the percentage of EtOH, thereby achieving a high degree of swelling for maximizing the QTP and QCT contents in the cHA hydrogels.

Figure 2B shows the effect of the amount of BDDE and the percentage of EtOH on the swelling ratio of hydrogels. HA concentration was established considering its dispersibility in 0.25 N NaOH solution and the final viscosity of HA dispersion for further cross-linking reaction. Except for 80% EtOH in the DW group, the swelling ratio of the cHA hydrogels prepared with a 1.1 weight ratio of BDDE to HA (BDDE/HA = 1.1, *w*/*w*) was higher than those with 2.2 and 3.3 ratios. High weight ratios of BDDE/HA (2.2 and 3.3) seem to attribute to make more compact hydrogel structures, which might be related to smaller pore size, with lower swelling ratios. In 80% EtOH in the DW group, different pattern of BDDE amount-dependent swelling ratio was shown due to its lower hydrophilicity than the other groups. Thus, the 1.1 weight ratio of BDDE to HA was chosen for cross-linking the HA hydrogels. The effect of the percentage of EtOH on the swelling ratios of the cHA hydrogels is shown in Figure 2C,D, where various percentages of EtOH mixed with either DW or PBS were used as swelling media. Although the swelling ratio of the cHA hydrogels was the highest in DW (0% EtOH), it was not suitable as a swelling solution due to the insufficient aqueous solubility of QTP and QCT. The over 80% EtOH (in both DW and PBS) groups were also excluded, since negligible swelling ratios of the cHA hydrogels were observed. It is notable that EtOH in the DW groups exhibited higher swelling properties than EtOH in the PBS groups (Figure 2C,D). The existence of pharmaceutical salts and different pH values seem to affect the swelling behavior. Thus, the QTP contents in the cHA hydrogels after swelling in various percentages of EtOH in a DW solution, followed by precipitation with PBS, were measured to optimize the solution for swelling the cHA hydrogels (Figure 2E). There was no significant difference in the QTP contents in the cHA hydrogels among the 50%, 60%, and 70% EtOH in the DW groups. However, when QCT (5 mg/mL) was added to the 50%, 60% and 70% EtOH in a DW solution, it was clearly solubilized only in the 70% EtOH solution (Figure S2). Thus, 70% EtOH in DW was selected as the hydration solution, and then the actual content of QCT in the various cHA hydrogels (cHA/QCT1, cHA/QCT3, and cHA/QCT5) was quantitatively determined after swelling and precipitation (Figure 2F). The content of QCT in the cHA hydrogels was linearly dependent on the loading amount of QCT, showing the highest QCT content in the cHA/QCT5 hydrogel. Therefore, the cHA/QTP/QCT5 hydrogel was finally selected for further evaluation, which was fabricated with 50 mg of HA, 55 mg of BDDE, 100 mg of the QTP base, and 50 mg of QCT. The cHA hydrogel was swollen for 24 h in an EtOH/DW (70:30, *v*/*v*) solution (10 mL) containing QTP and QCT, which was then precipitated by incubation in 20 mL of PBS for 24 h. It is notable that the final cHA/QTP/QCT5 hydrogel was transparent with a yellow color due to the QCT, and then changed to turbid by the formation of precipitates of QTP and QCT (Figure 1B). The actual contents of QTP and QCT in cHA/QTP/QCT5 were 16.4 ± 1.1 and 7.3 ± 0.9 μg/mg gel, respectively. For clinical application, the contents of QTP and QCT in developed hydrogels can be further controlled by altering the preparation procedures.

### 3.2. Physicochemical and Rheological Characterizations of the Hydrogels

**Figure 3:**
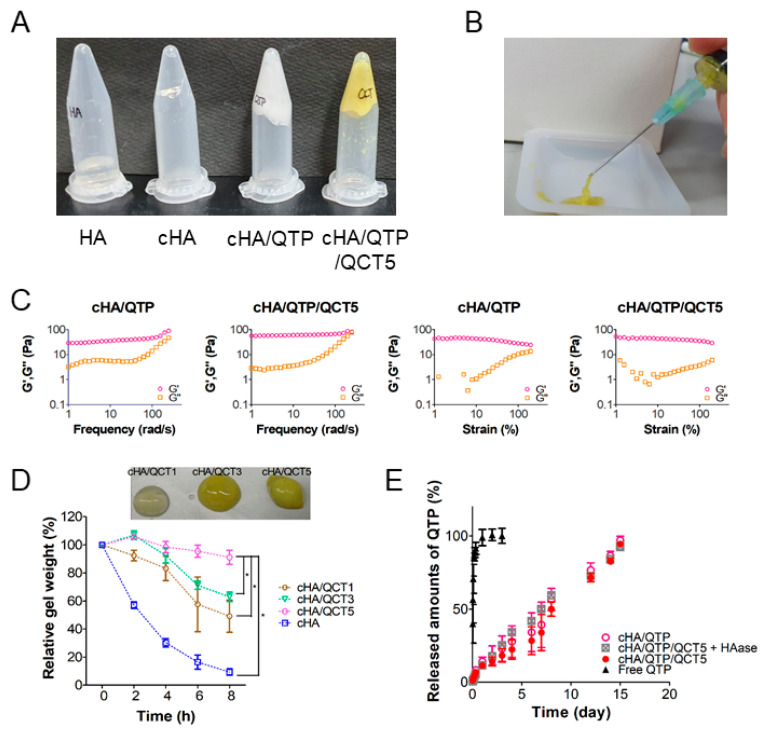
Rheological and physicochemical features of the cross-linked hydrogel formulations. (**A**) Gelation properties of the HA, cHA, cHA/QTP, and cHA/QTP/QCT5 hydrogels tested by an inversion assay. (**B**) Syringe injectability of the cHA/QTP/QCT5 hydrogel. (**C**) Frequency and strain-dependent viscoelastic properties of the cHA/QTP and cHA/QTP/QCT5 hydrogels. (**D**) In vitro degradation profiles of the cHA, cHA/QCT1, cHA/QCT3, and cHA/QCT5 hydrogels. The remaining weight percentage (%) of each hydrogel compared to the initial weight is plotted. Each point represents the mean ± SD (*n* = 3). (**E**) Dissolution profiles of QTP from the cHA/QTP and cHA/QTP/QCT5 (in the absence or presence of HAase) hydrogels. The hydrogel or QTP solution was loaded into a dialysis membrane sac, and was immersed into PBS containing 0.5% sodium lauryl sulfate (SLS). Each point represents the mean ± SD (*n* = 3).

The gelation behaviors of the optimized hydrogel formulations were investigated using an inversion test (Figure 3A) [27]. Compared to the pure HA group, the cHA group exhibited a halt in the cross-linked hydrogel mass in the inverse position. This implies the BDDE-initiated cross-linking of the HA dispersion and the elevation of its viscoelastic characteristics. 

Both the cHA/QTP and cHA/QTP/QCT5 specimens also stayed in the inverse position without flowing, indicating that the incorporation of QTP and QCT into the hydrogel system did not interfere with the BDDE-mediated cross-linking of the HA structure. The injectability of the cHA/QTP/QCT5 hydrogel was tested with a needle-connected plastic syringe (Figure 3B), which showed that it could easily pass through the 23-gauge syringe needle, implying that it is suitable for subcutaneous injection with minimal invasiveness. The viscoelastic properties of the cHA hydrogels (i.e., cHA/QTP and cHA/QTP/QCT5) were more precisely investigated using a rheometer (Figure 3C) [27]. Both the cHA/QTP and cHA/QTP/QCT5 hydrogels had higher G′ (storage modulus) value than G″ (loss modulus) values in the frequency and strain sweep data. The higher degree of elasticity compared to the degree of viscosity reflects the typical behaviors of viscoelastic solids. The retention of the cross-linked hydrogel fragments in the inverse position, as observed in the inversion assay (Figure 3A), can be further supported with these viscoelastic characteristics. Shear rate-dependent shear stress and viscosity data were also obtained (Figure S3). Decreasing viscosity pattern was shown with increasing shear rate, and it indicates the shear-thinning behavior of designed hydrogel. Observed shear-thinning behavior may help the easy injection of cHA/QTP/QCT5 hydrogel through the syringe needle to subcutaneous tissue.

The in vitro degradation profiles of the QCT-loaded cHA hydrogels (i.e., cHA/QCT1, cHA/QCT3, and cHA/QCT5) were determined to investigate the HAase inhibitory effect of QCT (Figure 3D). QCT was selected as an HAase inhibitor in this study for slowing down the degradation rate of the HA hydrogels. As shown in Figure 3D, the relative percentages of the remaining gel mass of the cHA, cHA/QCT1, cHA/QCT3, and cHA/QCT5 groups after 8 h of incubation was 9.3%, 49.2%, 63.0%, and 91.2%, respectively. While over 90% of the cHA hydrogels was disappeared for 8 h without QCT, QCT inhibited HAase in a dose-dependent manner, and thus over 90% of the cHA/QCT5 hydrogel was retained during the same period. These in vitro degradation data imply that the incorporation of QCT in cHA can efficiently retard the degradation rate of HA hydrogels.

Figure 3E shows the QTP release profiles of the cHA hydrogels. For attaining sink conditions for release tests, 0.5% SLS was solubilized in the release media. In the free QTP group, over 90% of the QTP was diffused from the dialysis membrane within 8 h, indicating that the dialysis membrane was not a barrier for the release. However, the cHA/QTP and cHA/QTP/QCT5 groups showed sustained release profiles and accomplished complete (nearly 100%) release of QTP on day 15. It is notable that no significant difference was shown in the release profiles of QTP between the cHA/QTP and cHA/QTP/QCT5 groups, indicating that QCT in the cHA did not affect the release of QTP, and the sustained release of QTP was accomplished over 15 days. The released amounts of QTP from cHA/QTP/QCT5 + HAase group were higher than cHA/QTP/QCT5 group on day 3–8, however, there was no significant difference in drug release rate between those two groups on day 15 probably due to the complete drug release. Drug release mechanisms were elucidated by mathematical modeling (Table S1). In a cylinder geometry, *n* = 0.45, 0.45 < *n* < 0.89, *n* = 0.89, and *n* > 0.89 indicate Fickian diffusion, anomalous transport, Case II transport, and Super Case II transport, respectively [32,33]. Considering calculated *n* values of all experimental groups, three hydrogel groups (cHA/QTP, cHA/QTP/QCT5, and cHA/QTP/QCT5 + HAase) exhibited non-Fickian processes. Of note, cHA/QTP/QCT5 + HAase group had an anomalous transport mechanism, which had similar degree of solvent diffusion and polymeric relaxation. Enzymatic degradation of cHA/QTP/QCT5 hydrogel by HAase induced those dual drug release mechanisms.

### 3.3. Biodegradation

**Figure 4:**
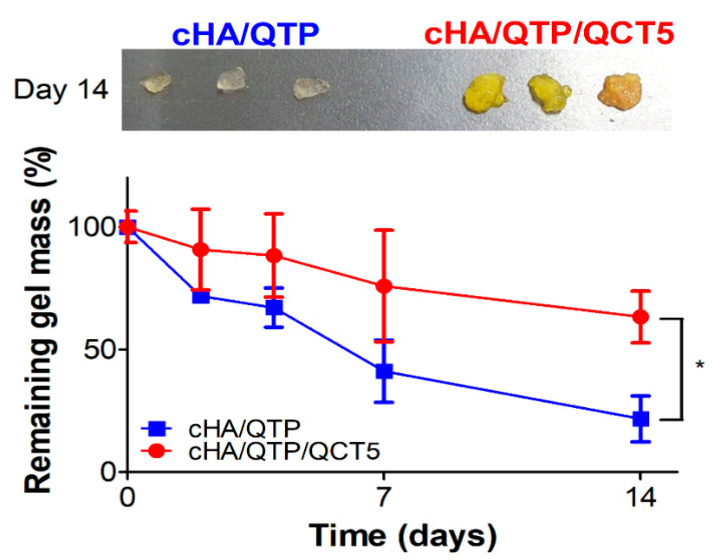
In vivo degradation profiles of the cHA/QTP and cHA/QTP/QCT5 hydrogels following subcutaneous injection in mice, and images of dissected hydrogels after 14 days. The remaining weight percentage (%) of the hydrogels compared to the initial weight is plotted. Each point represents the mean ± SD (*n* = 3).

The in vivo degradation profiles of the cHA/QTP and cHA/QTP/QCT5 hydrogels were investigated after subcutaneous injection in mice (Figure 4). The remaining weight of the cHA/QTP hydrogel decreased more rapidly than that of cHA/QTP/QCT5. The relative remaining weight of the cHA/QTP hydrogel (22%) was significantly lower than that of cHA/QTP/QCT5 (63%) on day 14 (*p* < 0.05), which matches the in vitro degradation data (Figure 3D). The slower degradation rate of cHA/QTP/QCT5, compared to cHA/QTP, supports the inhibitory effect of QCT on HAase. Thus, the incorporation of an HAase inhibitor could further extend the retention of a cHA/QTP hydrogel in the injection site, thereby contributing to the sustained release of the incorporated drug.

### 3.4. In Vivo Toxicity of the Cross-Linked Hydrogels

**Figure 5:**
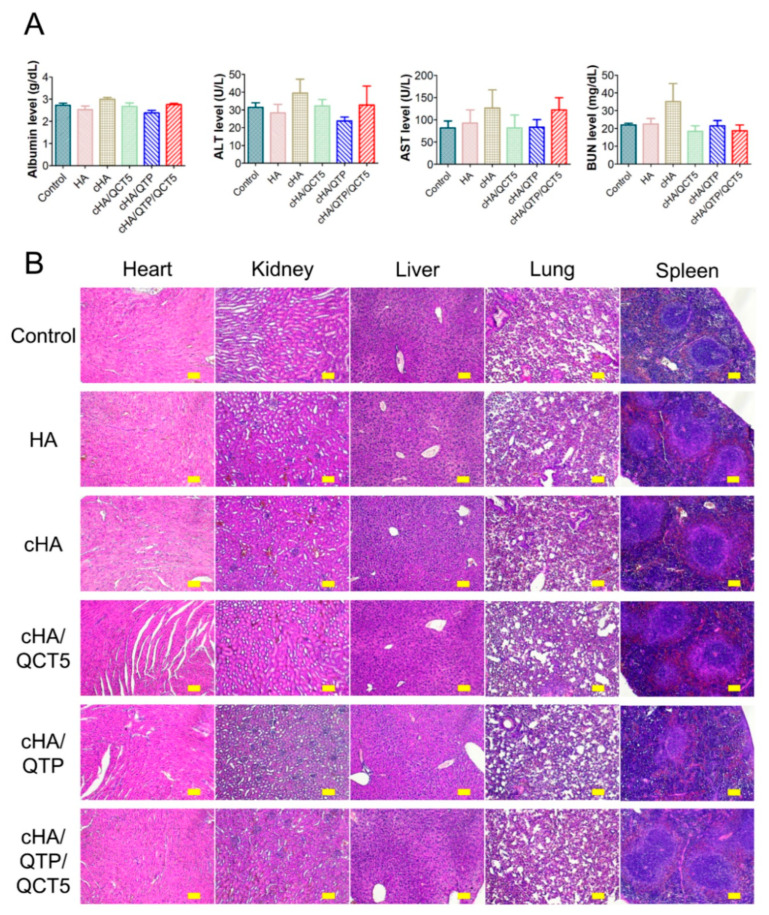
In vivo toxicity of the hydrogels (HA, cHA, cHA/QCT5, cHA/QTP, and cHA/QTP/QCT5) in mice after 7 days of subcutaneous injection. (**A**) Blood chemistry data. Each point represents the mean ± SD (*n* = 4). (**B**) Hematoxylin and eosin (H&E) staining images. The length of the scale bar is 100 μm. ALT, alanine transaminase; AST, aspartate transaminase; BUN, blood urea nitrogen.

The in vivo toxicities of the cHA hydrogel formulations were investigated on day 7 after subcutaneous injection in mice. The blood chemistry data and H&E staining images of the HA, cHA, cHA/QCT5, cHA/QTP, and cHA/QTP/QCT5 groups are shown in Figure 5. It is notable that there were no significant differences in the albumin, ALT, AST, and BUN levels among the control and cHA/QTP/QCT5 hydrogel groups (Figure 5A). Moreover, there were no significant differences in the H&E staining images of the cHA/QTP/QCT5 group compared to the control (Figure 5B). These data imply that the subcutaneous injection of cHA hydrogel formulations in mice does not induce severe toxicities to tested organs and tissues. 

HA is known to be a biocompatible and biodegradable polymer, and thus, it has been widely utilized for biomedical applications [7]. As diverse BDDE-cross-linked HA hydrogels have also been applied in dermal fillers, they can easily be used in clinics for subcutaneous injection by controlling the BDDE concentration [16,25]. It is known that an ether bond of cHA can be cleaved by cytochrome P450, while BDDE is hydrolyzed to glycerol and butanediol, after which it is eliminated via urine [18]. Although a low degree of BDDE cross-linking could reduce the possible toxicities of HA-based hydrogels, it may also result in a short retention time in subcutaneous tissue by rapid degradation after injection. Therefore, the incorporation of QCT, as an HAase inhibitor, in cHA hydrogels can be an alternative strategy to extend the retention time, while minimizing the toxicities related to BDDE cross-linking.

### 3.5. In Vivo Pharmacokinetics

**Figure 6:**
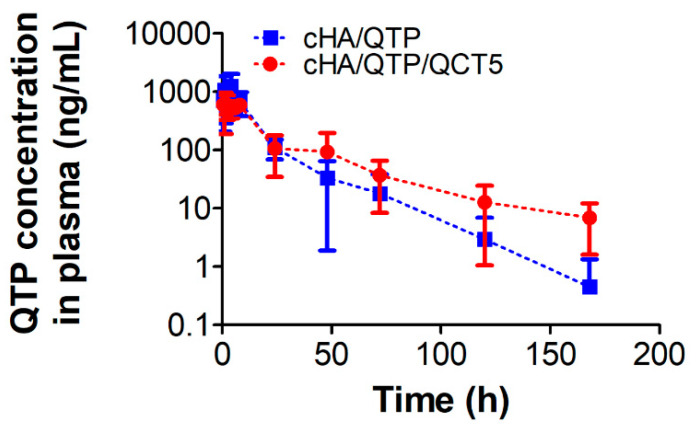
Plasma concentration profiles of QTP after subcutaneous injection of the cHA/QTP or cHA/QTP/QCT5 hydrogels (20 mg/kg as QTP) in rats. Each point represents the mean ± SD (*n* = 4).

The QTP concentration profiles in the plasma after subcutaneous injection of the cHA hydrogel formulations (20 mg/kg as QTP) in rats are plotted in Figure 6, and the pharmacokinetic parameters are summarized in Table 1. Since the biological half-life of QTP in rats (following intragastric administration) has been reported to be 2.1 h [34], it is notable that the cHA hydrogel groups showed slow elimination profiles regarding the QTP plasma concentration for up to 7 days. Moreover, it is interesting to report that the cHA/QTP/QCT5 group showed a slower elimination profile than that of the cHA/QTP group. There was no significant difference in the AUC and T_max_ of the cHA/QTP and cHA/QTP/QCT5 groups (*p* > 0.05) (Table 1), However, the C_max_ of cHA/QTP was significantly higher than that of the cHA/QTP/QCT5 group, while the t_1/2_ and MRT of the cHA/QTP/QCT5 group were higher than those of the cHA/QTP group (*p* < 0.05). This indicates that the incorporation of QCT into cHA hydrogel formulations can successfully prolong the release of QTP with a lower initial burst release. Figure 3E shows that the in vitro release of QTP was not affected by the addition of QCT in the cHA hydrogels, while the in vitro and in vivo degradation of cHA was inhibited (Figure 3D and Figure 4). Thus, the addition of QCT to the cHA hydrogels seemed to successfully inhibit the HAase activity and contributed to the sustained release of QTP by retarding the degradation of the cHA hydrogels.

## 4. Conclusions

The composition and preparation of cHA hydrogels with minimum BDDE cross-linking were optimized to ensure a high swelling ratio and drug content. An HAase inhibitor (i.e., QCT) was incorporated in the cHA hydrogels to retard the rapid degradation of cHA. QCT and QTP were successfully incorporated by the antisolvent precipitation method. The optimized cHA hydrogels had suitable rheological properties for subcutaneous injection and did not show any obvious toxicities. The results of the pharmacokinetic study after the subcutaneous injection of the cHA hydrogels in rats indicated that the incorporation of QCT in the cHA hydrogel formulations successfully prolonged the release of QTP with a lower initial burst release. The retarded degradation of the cHA hydrogels confirmed in the in vitro and in vivo studies could be contributed to the sustained release of QTP. Taken together, the incorporation of HAase inhibitors in cHA hydrogels can extend their retention in subcutaneous tissue and offer a suitable strategy to prolong the release of incorporated drugs. They can be a novel approach to improve patient compliance by reducing dosing frequency, and thus, it would be promising to investigate their translational feasibility for clinical applications.

## Figures and Tables

**Table 1:** Pharmacokinetic parameters of quetiapine (QTP) following subcutaneous injection in rats.

Parameter	cHA/QTP	cHA/QTP/QCT5
AUC (ng∙h/mL)	15,772.0 ± 5439.3	15,875.1 ± 3531.2
C_max_ (ng/mL)	1827.6 ± 481.3	782.6 ± 174.4 *
T_max_ (h)	2.4 ± 1.9	3.0 ± 3.4
t_1/2_ (h)	13.4 ± 4.9	23.5 ± 2.7 *
MRT (h)	14.3 ± 4.8	30.9 ± 3.9 *

* *p* < 0.05, compared to the cHA/QTP group. The data are presented as the mean ± SD (*n* = 4). AUC, total area under the plasma concentration–time curve from time zero to infinity; C_max_, the peak plasma concentration; T_max_, the time to reach C_max_; t_1/2_, half-life; MRT, mean residence time.

## Data Availability

The data presented in this study are available on request from the corresponding author. The data are not publicly available due to intellectual properties-related issues.

## Supplementary Materials

The following are available online at https://www.mdpi.com/1999-4923/13/2/170/s1, Figure S1: Synthesis of HA-BDDE (cHA) conjugate, Figure S2: Images of QCT dispersion (at 5 mg/mL) in 50%, 60%, and 70% EtOH (in DW), Figure S3: Shear rate-dependent shear stress and viscosity profiles of cHA/QTP/QCT5 hydrogel, Table S1: Korsmeyer-Peppas modeling of release data of QTP.

## Abbreviations

ALT	Alanine transaminase
AST	Aspartate transaminase
AUC	Total area under the plasma concentration–time curve from time zero to infinity
BDDE	1,4-Butanediol diglycidyl ether
BUN	Blood urea nitrogen
cHA	Cross-linked hyaluronic acid
C_max_	Peak plasma concentration
DW	Distilled water
EtOH	Ethanol
HA	Hyaluronic acid
HAase	Hyaluronidase
HPLC	High-performance liquid chromatography
IS	Internal standard
MRT	Mean residence time
PBS	Phosphate-buffered saline
PEG	Polyethylene glycol
QCT	Quercetin
QTP	Quetiapine
T_max_	Time to reach C_max_
