# Supplementary material for: Hyaluronidase Inhibitor-Incorporated Cross-Linked Hyaluronic Acid Hydrogels for Subcutaneous Injection
Source: Pharmaceutics. 2021 Jan 27;13(2):170. doi: 10.3390/pharmaceutics13020170 (PMC7910999; doi:10.3390/pharmaceutics13020170)
Supplement: Supplementary file 1 [file pharmaceutics-13-00170-s001.pdf]

pharmaceutics

MDPI

Supplementary Materials

Hyaluronidase Inhibitor-Incorporated Cross-Linked Hyaluronic Acid Hydrogels for Subcutaneous Injection

Min-Hwan Kim, Ju-Hwan Park, Duy-Thuc Nguyen, Sungyun Kim, Da In Jeong, Hyun-Jong Cho and Dae-Duk Kim

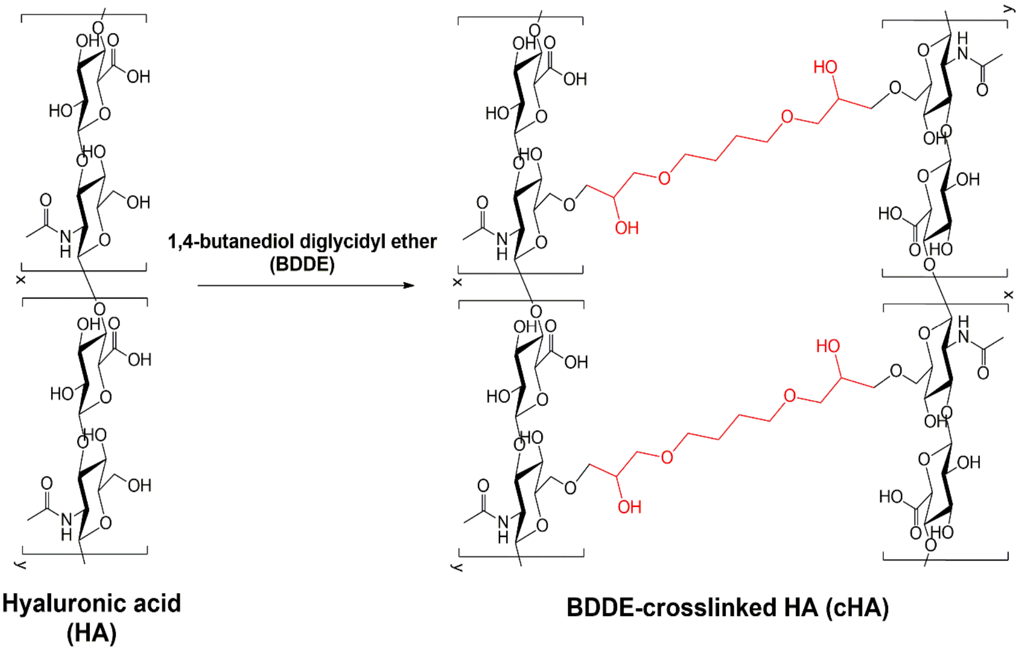

Figure S1. Synthesis of HA-BDDE (cHA) conjugate.

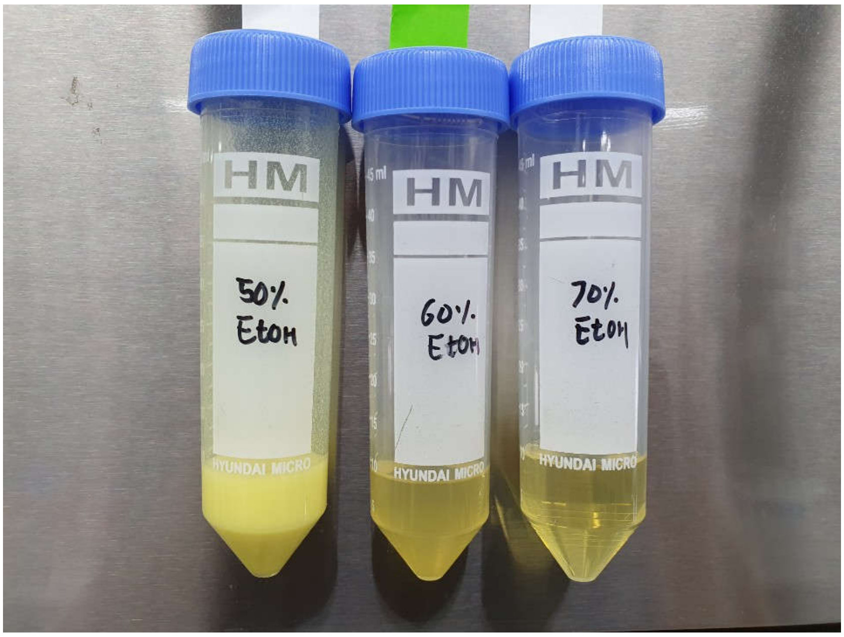

Figure S2. Images of QCT dispersion (at 5 mg/mL) in 50%, 60%, and 70% EtOH (in DW).

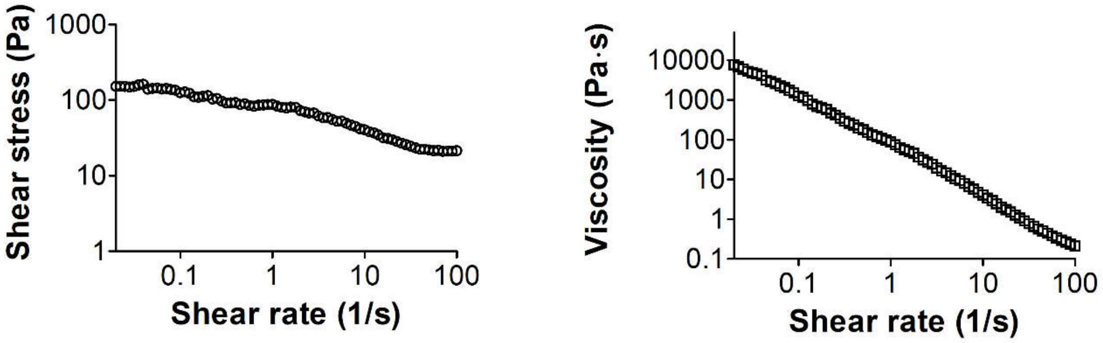

Figure S3. Shear rate-dependent shear stress and viscosity profiles of cHA/QTP/QCT5 hydrogel.

Table S1. Korsmeyer-Peppas modeling of release data of QTP.

Group	R^2	k_{KP}	n
Free QTP	0.7126	62.75	0.1311
cHA/QTP	0.9540	0.904	0.973
cHA/QTP/QCT5	0.9534	0.348	1.086
cHA/QTP/QCT5 + HAase	0.9952	1.062	0.775

Korsmeyer-Peppas equation: $\mathrm{F} = k_{KP} \times t^n$
